# Guillain-Barre Syndrome Associated With COVID-19 Infection: A Case Report With Review of Literature

**DOI:** 10.7759/cureus.13096

**Published:** 2021-02-03

**Authors:** Mostafa Meshref, Hessa A. Alshammari, Shiamaa M Khairat, Roohi Khan, Imran Khan

**Affiliations:** 1 Neurology, Al-Azhar University, Cairo, EGY; 2 Internal Medicine, King Khaled Hospital, Hail, SAU; 3 Neurology, King Khaled Hospital, Hail, SAU

**Keywords:** covid-19, guillain-barré syndrome, acute motor axonal neuropathy

## Abstract

COVID-19 has become a pandemic. It affects multiple systems of the body including the nervous system. It invades the nervous systems through multiple routes - either olfactory tract, bloodstream (by binding to endothelial receptors) or via ACE-2 receptors in the brain. We report a case of Guillain-Barré syndrome (GBS) variant (acute motor axonal neuropathy (AMAN type)) associated with COVID-19 infection with positive polymerase chain reaction (PCR) test for COVID-19 and positive contact history with infected family member. GBS and its variants like AMAN can occur due to COVID-19 infection through its immune-mediated effects. Diagnosis of GBS should depend on the clinical and supportive criteria. The treatment should be started early to prevent progression and disease co-morbidities.

## Introduction

Severe acute respiratory syndrome coronavirus 2 (SARS-CoV-2) has become pandemic for the last few months worldwide. It is caused by a group of enveloped non-segmented positive-sense RNA viruses called Coronavirus [1]. COVID-19 infection not only affects respiratory system, but also can affect various body systems such as neurological (either central or peripheral), cardiac, gastrointestinal and skin [2,3]. Multiple proposed hypotheses have explained that COVID-19 infection transmits to the central nervous system through olfactory tract, bloodstream (by binding to endothelial receptor) or through ACE-2 receptors in the brain [4]. The central nervous system manifestations such as dizziness, encephalitis, encephalopathy, cerebrovascular accident (CVA), anosmia, ageusia, epilepsy, ataxia and cerebral haemorrhage are the most prevalent occurrence among COVID-19 patients which strengthens the methods of neurotropism of coronavirus [4]. However, peripheral nervous system (PNS) can also be affected with several manifestations such as musculoskeletal system affection and Guillain-Barré syndrome (GBS) [5]. GBS represents the most common cause of acute flaccid paralysis [6]. GBS is an autoimmune disorder related to the peripheral nervous system. The clinical characteristics of GBS are progressive weakness of the limbs and reduction in or loss of tendon reflexes (hyporeflexia and areflexia, respectively). In this disorder, protein concentrations in the cerebrospinal fluid (CSF) increase, while the white cell count is normal [7]. The clinical GBS variants include a classic sensorimotor type, Miller Fisher syndrome (MFS), bilateral facial palsy with paraesthesia, pure motor, pure sensory, paraparetic, pharyngeal-cervical-brachial variants, polyneuritis cranialis (GBS-MFS overlap), and Bickerstaff brainstem encephalitis [6].

The pathogenesis of peripheral nerve damage can be linked to triggering of an abnormal response of immune system to variants of infectious conditions, which can in some cases lead to the production of antibodies autoreactivity (anti-ganglioside antibodies), where the immune system is activated and starts attacking body's own neurons [6]. The most common microbes that cause GBS include Campylobacter jejuni, Epstein-Barr virus, cytomegalovirus, influenza virus and mycoplasma. The other coronavirus (SARS-CoV and MERS) and Zika virus have also been associated with GBS [8]. Therefore, since the start of pandemic, many cases with close association between COVID-19 and GBS incidence have been reported worldwide [9]. The pathogenesis of COVID-19 and GBS (or other immune neurological conditions) can be linked to systemic inflammation and immune dysregulation mechanism in which there is a maladaptive immune response characterized by hyperactivity of innate immunity followed by immunosuppression [10].

## Case presentation

**Figure 1:**
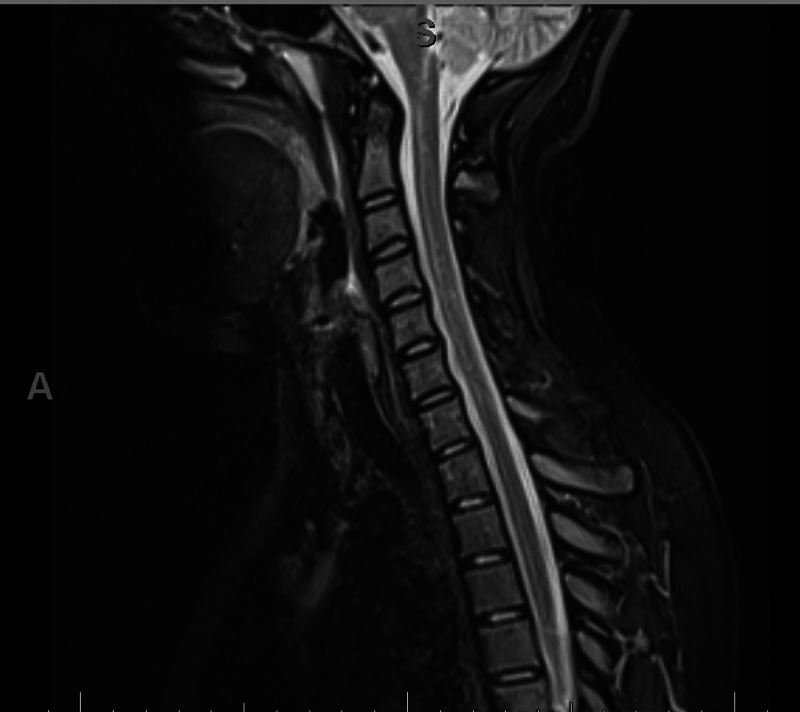
MRI cervical spine without contrast

**Figure 2:**
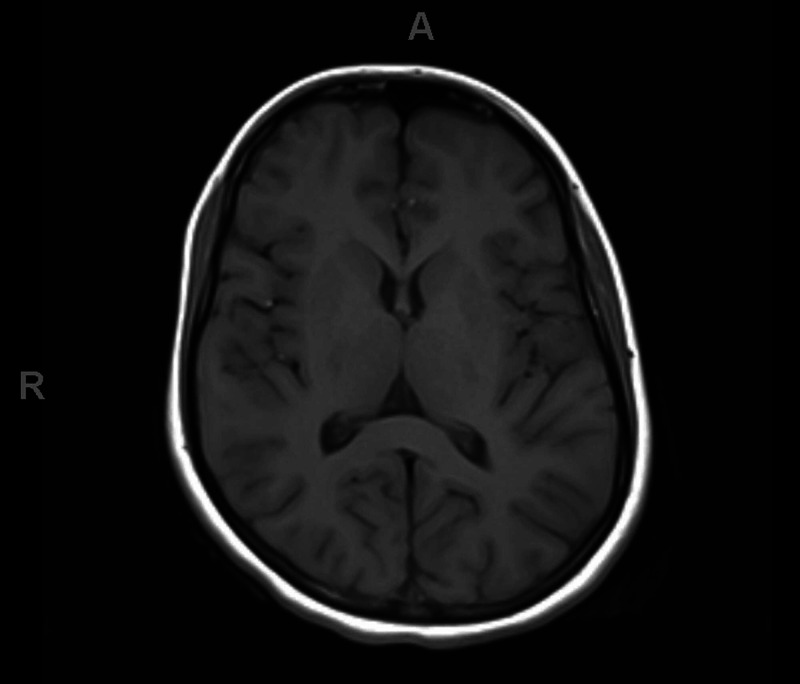
MRI brain

**Figure 3:**
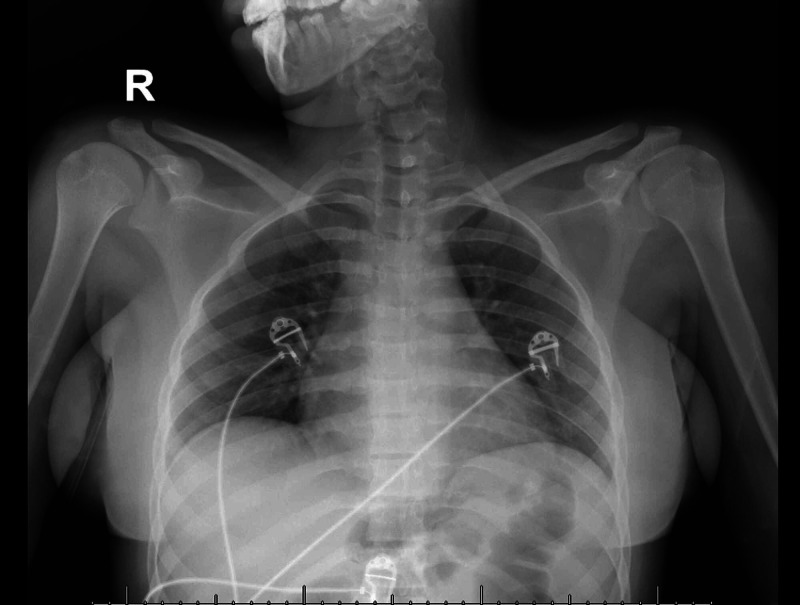
X-ray chest

An 18-year-old patient with no co-morbidity presented to our hospital. She complained of acute onset of inability to stand from sitting position for four days which was associated with bilateral lower limb weakness and difficulty in swallowing for two days. There was no history of preceding infectious conditions like upper respiratory tract or gastrointestinal symptoms. On examination, the patient was fully conscious, alert and oriented. Her speech had nasal tone with facial diplegia and intact gag reflex and central uvula. The motor system examination showed quadriparesis of both upper and lower limbs with equal grade of weakness (grade 3 on MRC scale). The weakness distribution was proximal, more than distal in both upper and lower limbs with generalized hyporeflexia and downgoing plantar response bilaterally. All sensory modalities were intact. The patient was walking aided by her family member, so gait could not be examined at that time. Thus, depending on the history, clinical scenario and examination, the provisional diagnosis was GBS. The patient was admitted to the ICU and started on intravenous immunoglobulin (400 mg/kg for five days) and prophylactic anticoagulant (for deep venous thrombosis (DVT) prophylaxis) with monitoring of vital signs. Also, we ordered routine labs including creatine phosphokinase (CPK), erythrocyte sedimentation rate (ESR), C-reactive protein (C-RP) (results were normal), MRI cervical (Figure 1) and MRI brain (Figure 2) (their result came normal). The patient had contact with one of her family members (her aunt) who had been diagnosed as COVID-19 positive one week before the starting of her condition. The COVID-19 swab (nasopharyngeal swab) was taken and result was positive, however, her chest X-ray was normal (Figure 3). The patient and her family refused for lumbar puncture and was planned for nerve conduction studies after finishing the treatment course (as it is not available at our hospital). The patient’s condition improved with the treatment course (five days of hospital stay) - i.e., improvement in weakness, difficulty swallowing and nasal tone of speech.

## Discussion

We are presently in a pandemic dealing with a virus called severe acute respiratory syndrome coronavirus 2 (SARS-CoV-2) that first originated as an outbreak of a respiratory illness in Wuhan City, China, in December 2019 [1]. COVID-19 infection not only affects the respiratory system, but also affects multiple body systems including neurological (central or peripheral), cardiac, gastrointestinal and skin as discussed in many studies before [3]. COVID-19 infection is transmitted to the nervous system via, according to multiple proposed hypothesis, olfactory tract, bloodstream (by binding to endothelial receptors) or via ACE-2 receptors in the brain [4]. The peripheral nervous system manifestations can occur with COVID-19 infection such as cranial nerve involvement (loss of smell, loss of taste and Bell's palsy) and Guillain-Barré syndrome and its variants as discussed in previous studies [10-13]. GBS is an inflammatory polyradiculoneuropathy which is also associated with numerous viral infections. Recently, there have been many case reports describing the association between COVID-19 and GBS [14]. Our patient had classic clinical picture of progressive symmetrical quadriparesis of lower motor neuron (LMN) distribution with acute onset (within three days) and absence of deep tendon reflexes and intact sensation with supportive criteria of bilateral facial nerve weakness of LMN character. Also, other complications like spinal cord lesion (e.g., myelitis or spinal cord compression) and brain lesions (e.g., stoke or parasagittal space-occupying lesion) were excluded through MRI cervical and MRI brain, and with mentioned clinical presentation this supports the diagnosis of GBS as discussed in Leonhard et al. [6].

The GBS and its variants can occur with COVID-19 due to the stimulatory effect of COVID-19 on the immune system and production of immune-mediated processes through stimulation of various inflammatory cells and cytokine production [10]. There are also multiple studies which hypothesize that angiotensin-converting enzyme 2 (ACE2) acts as a functional receptor of SARS-CoV-2 in human tissues. Considering the similarity in the sequencing of the SARS-CoV and SARS-CoV-2 spike proteins, it has been suggested that SARS-CoV-2 also uses ACE2 as a functional receptor and through this can invade the PNS and cause GBS as mentioned in Hamming et al. [15], Wu et al. [16] and Letko et al. [17]. There is another possible hypothesis, which needs more investigation, that states that COVID-19 might produce antibodies against specific gangliosides that usually appear with certain forms of GBS as mentioned by Sedaghat and Karimi [18]. The majority of GBS-associated COVID-19 depends on polymerase chain reaction (PCR) test to diagnose the existence of COVID-19 infection as discussed in Esteban Molina et al. [19] and Trujillo Gittermann et al. [20]. The response of the case to immune therapy (intravenous immunoglobulin) elevates the possibility of immune-mediated response of COVID-19 and its impact on GBS occurrence. Thereby, this can give hope in immune therapy and immunomodulatory drugs to be used in the treatment of this infection which will need more investigations and more clinical trials.

## Conclusions

GBS-associated COVID-19 infection is common. Thus, it needs to be early managed if clinically suspected. Contact history of diseased family member should be suspected and asked for every patient in this pandemic era.
